# Polymorphisms associated with type 2 diabetes in familial longevity: The Leiden Longevity Study

**DOI:** 10.18632/aging.100250

**Published:** 2010-12-20

**Authors:** Simon P. Mooijaart, Diana van Heemst, Raymond Noordam, Maarten P. Rozing, Carolien A. Wijsman, Anton J.M. de Craen, Rudi G.J. Westendorp, Marian Beekman, Eline P. Slagboom

**Affiliations:** ^1^ Department of Gerontology and Geriatrics, Leiden University Medical Centre, Leiden, The Netherlands; ^2^ Section of Molecular Epidemiology of the Department of Medical Statistics, Leiden University Medical Centre, Leiden, The Netherlands; ^3^ The Netherlands Consortium for Healthy Aging, The Netherlands

**Keywords:** Diabetes Mellitus, polymorphism, SNP, glucose, insulin, longevity

## Abstract

Human longevity is in part genetically determined, and the insulin/IGF-1 signal transduction (IIS) pathway has consistently been implicated. In humans, type 2 diabetes is a frequent disease that results from loss of glucose homeostasis and for which new candidate polymorphisms now rapidly emerge from genome wide association studies.

In the Leiden Longevity Study (n=2415), the offspring of long lived siblings (“offspring”) who are genetically enriched for longevity were shown to have a more beneficial metabolic profile compared to their environmentally matched partners (“controls”), including better glucose tolerance. We tested whether the “offspring” carry a lower burden of diabetes risk alleles. Fifteen polymorphisms derived from genome wide association (GWA) scans in type 2 diabetes were tested for association with parameters of glucose metabolism in offspring and controls, and burden of risk alleles was compared between offspring and controls.

Among all participants, a higher number of type 2 diabetes risk alleles associated with a higher prevalence of diabetes (P=0.011) and higher serum concentration of glucose (P<0.016) but not insulin (P=0.450). None of the polymorphisms differed in frequency between the offspring and controls (all P>0.05), nor did the mean total number of risk alleles (P=0.977). The association between polymorphisms and glucose levels did not differ between controls and offspring (Pinteraction=0.523).

The better glucose tolerance of the “offspring” is not explained by a lower burden of type 2 diabetes risk alleles, suggesting that specific mechanisms determining longevity exist.

## INTRODUCTION

Human longevity is characterized at middle age by lower prevalence of myocardial infarction, hypertension and type 2 diabetes [1]. Also, middle aged offspring of long-lived families exhibit lower plasma levels of glucose and higher insulin sensitivity. This is in concordance with the findings from animal studies which revealed that the insulin/IGF1 signal transduction pathway is involved in lifespan (reviewed in [2]). In humans, using a candidate driven approach, we and others have shown that genetic variation in this pathway affects human longevity [3, 4].

Type 2 diabetes (T2D) is characterized by an increased insulin resistance. In humans, insulin resistance as well as the prevalence of T2D increases with age. Long-lived subjects, such as centenarians and nonagenarian siblings, as well as their offspring, were found to exhibit a remarkably decreased prevalence of type 2 diabetes. Recently, offspring of longlived siblings were also found to have better glucose tolerance and higher insulin sensitivity as determined by homeostatic model assessment [5]. Since increased insulin sensitivity is associated with longevity, genetic determinants of T2D may be of interest for studies on longevity. In the last years, genome wide association (GWA) studies have identified several polymorphisms that associate with increased risk of T2D [6-9]. Replication studies [10-12] have shown the clinical relevance of a number of the identified loci.

To investigate whether the better glucose tolerance phenotype in the offspring of long-lived individuals is due to lack of genetic variants associated with type 2 diabetes, we analyzed 15 well established type 2 diabetes variants in the Leiden Longevity Study [13] for their association with familial longevity. The objective of the present study was to investigate whether a lower burden of common genetic variants that have been associated with increased T2D in GWA studies can account for the beneficial glucose tolerance associated with familial longevity.

**Table 1.** Information of the fifteen selected SNPs associated with type 2 diabetes in Genome Wide Association studies

SNP	Gene/locus	Location	Risk allele	References
Rs10497721	*TMEFF2*	2q32.3	A/C	[21]
Rs1801282	*PPARG*	3p25	C/G	[21], [22]
Rs4402960	*IGF2BP2*	3q27.2	T/G	[22], [23]
Rs10010131	*WFS1*	4q16	G/A	[24], [23]
Rs7754840	*CDKAL1*	6p22.3	C/G	[23], [12]
Rs13266634	*SLC30A8*	8q24.11	C/T	[23], [10]
Rs564398	*CDKN2A/2B*	9q21	T/C	[25], [12]
Rs10811661	*CDKN2A/2B*	9q21	T/C	[10], [22]
Rs1111875	*HHEX*	10q23	C/T	[26], [23]
Rs7903146	*TCF7L2*	10q25.2	T/C	[27], [10]
Rs5219	*KCNJ11*	11p15.1	T/C	[28], [29]
Rs1495377	*TSPAN8*	12q21.1	T2D	[9]
Rs8050136	*FTO*	16q12.2	A/C	[23], [30]
rs4430796	*HNF1B*	17q12	A/G	[31], [32]
rs757210	*HNF1B*	17q12	T/C	[31]

Abbreviations: TMEFF2, transmembrane protein with EGF-like and 2 follistatin like domains 2; PPARG, peroxisome proliferator activated receptor γ; IGFBP2, IGF binding protein 2; WFS1, Wolfram Syndrome 1; CDKAL1, CDK5 reg. sub. Ass. protein 1; SLC30A8, solute carries family 30; CDKN2A/2B, cyclin-dependent kinase inhibitor 2A/2B; HHEX, Hematopoietically expressed Homeobox; TCF7L2, transcription factor 7 like 2; KCNJ11, Potassium channel inwardly rectifying submfamily J member 11; TSPAN8, tatraspanin8; FTO, fat mass and obesity associated; HNF1B, HNF1 homeobox B

## RESULTS

### Baseline characteristics

The total study population consisted of 2415 participants (1671 offspring; 744 controls). Baseline characteristics are shown in Table 2. The group of offspring was slightly older compared to their partners. The two groups were comparable with respect to measures of height, weight and body mass index, both crude and after adjustment for age and sex. Offspring from nonagenarian siblings had a lower prevalence of diabetes mellitus and hypertension as well as a slightly lower prevalence of myocardial infarction. In accordance with the lower prevalence of diabetes, offspring had lower glucose levels (*P*<0.001) and lower levels of insulin (*P*=0.006). After exclusion of all participants with diabetes, the association remained significant for glucose (*P*=0.001), but became non-significant for insulin (*P*=0.209).

**Table 2.** Baseline characteristics of the study groups from the Leiden Longevity Study

	Offspring (n = 1671)	Controls (n = 744)	P-Value
**Demographics**			
Age in years, mean (SD)	59.4 (6.5)	58.7 (7.5)	0.032
Females, number (%)	900 (54%)	429 (58%)	0.083
**Antropometrics**			
Height (cm), mean (95% CI)	172.9 (172.5–173.2)	172.9 (172.3–173.4)	0.951
Weight (kg), mean (95% CI)	76.1 (75.4–76.8)	76.8 (75.8–77.8)	0.279
Body mass index (kg/m2), mean (95% CI)	25.4 (25.2–25.6)	25.6 (25.3–25.9)	0.268
**Disease prevalence**			
Diabetes Mellitus, number (%)	60 (4%)	47 (7%)	0.003
Hypertension, number (%)	319 (23%)	179 (28%)	0.003
Myocardial infarction, number (%)	33 (2%)	25 (4%)	0.040
Stroke, number (%)	46 (3%)	18 (3%)	0.602
**Glucose metabolism**			
Glucose (mmol/L), mean (95% CI)	5.75 (5.70–5.81)	6.01 (5.92–6.09)	<0.001
Insulin (mU/L), geometric mean (95% CI)	16.1 (15.5–16.8)	18.0 (16.9–19.1)	0.006

### Association of T2D risk alleles with diabetes and glucose levels

We observed an increasing prevalence of diabetes mellitus with an increasing number of T2D risk alleles (*P* = 0.011, Table 3). We also tested the association between numbers of risk alleles and serum parameters of glucose metabolism in the total group. With increasing numbers of risk alleles, we found an increase in glucose (*P*= 0.016) but not insulin (*P*=0.450). We found that the number of risk alleles was not associated with body mass index (BMI), and repeating the analyses with adjustment for BMI did not materially change the results (data not shown). After exclusion of participants with DM, statistical significance was lost for the association of number of alleles with levels of glucose (*P* = 0.089). In the oral glucose tolerance test increasing number of alleles associated with increasing area under the curve for glucose (*P* = 0.018).

### Allele frequencies in partners and offspring

Next we tested the hypothesis that differences in allele frequencies of these SNPs could explain the observed difference in prevalence of DM and differences in glucose and insulin levels between offspring and controls (Table 4). For none of the SNPs, the allele frequency was significantly different between offspring from familial nonagenarians compared to their partners. Likewise, no differences were found in the mean number of T2D risk alleles between the groups of offspring and controls (14.5 vs. 14.5 respectively, *P*=0.977).

### Interaction

**Figure 1.**
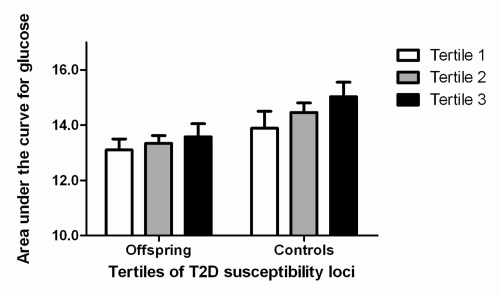
Association between increasing number of type 2 diabetes susceptibility loci, partitioned according to tertiles, and area under the curve for glucose. T2D: type 2 diabetes. Results were adjusted for sex and age. Number of participants per tertile for group of offspring: first tertile (n = 44), second tertile (n = 38), third tertile (n = 29). Number of participants per tertile for group of controls: first tertile (n = 33), second tertile (n = 38), third tertile (n = 34).

To assess whether the offspring of nonagenarian sibling pairs were more protected against the influences of the risk alleles than the controls, we compared the increase in glucose dependent on the number of risk alleles in the offspring and controls. When analyzing offspring and controls separately, levels of glucose increased with an increasing number of number of risk alleles in offspring (*P*=0.016) and also in controls, albeit not significantly (*P*= 0.369). The increase in the control group did not reach statistical significance, possibly because of the smaller size of the group. The increase in glucose levels dependent on number of type 2 diabetes risk alleles was not different between offspring and partners (*P* for interaction = 0.538). A similar finding was found for the area under the curve in the oral glucose tolerance test (Figure 1). In both offspring and controls a similar trend was seen, albeit not statistically significant (*P* = 0.093 and *P* = 0.159 respectively) due to small samples size. There was no significant interaction (*P* = 0.797).

**Table 3.** Association of the number of risk alleles associated with type 2 diabetes in offspring and partners combined

	Stratum of number of risk alleles			P for trend
	5-13 (n=731)	14-15 (n=647)	16-23 (n=712)	
**Demographics**				
Age in years, mean (SD)	59.2 (6.7)	59.1 (6.8)	59.6 (7.0)	0.541
Females, number (%)	397 (54%)	359 (56%)	393 (55%)	0.981
**Antropometrics**				
Height (m), mean (95% CI)	172.6 (172.1-173.1)	173.1 (172.6-173.7)	173.0 (172.4-173.5)	0.354
Weight (kg), mean (95% CI)	76.0 (75.0-77.0)	76.5 (75.5-77.5)	76.3 (75.3-77.3)	0.446
Body mass index (kg/m2), mean (95% CI)	25.5 (25.2-25.8)	25.5 (25.2-25.8)	25.4 (25.1-25.7)	0.859
**Disease prevalence**				
Diabetes Mellitus, number (%)	24 (3%)	28 (4%)	39 (6%)	0.011
Hypertension, number (%)	140 (19%)	124 (21%)	153 (22%)	0.188
Myocardial infarction, number (%)	21 (3%)	12 (2%)	19 (3%)	0.133
Stroke, number (%)	20 (3%)	16 (3%)	19 (3%)	0.194
**Glucose metabolism**				
Glucose (mmol/L), mean (95% CI)	5.78 (5.70-5.87)	5.79 (5.69-5.88)	5.90 (5.82-5.99)	0.016
Insulin (mU/L), geometric mean (95% CI)	16.9 (16.0-18.0)	16.6 (15.5-17.6)	16.3 (15.3-17.5)	0.450

P-values were calculated with the number of risk alleles as continuous variable, adjusting for age and sex, and using robust standard errors to account for family relationships among the offspring.

**Table 4.** Comparison of allele frequencies and number of risk alleles associated with type 2 diabetes in offspring and controls

	Offspring (n = 1671)	Controls (n = 744)	P-Value
Rs10497721	0.09	0.10	0.918
Rs1801282	0.87	0.88	0.363
Rs4402960	0.29	0.31	0.355
Rs10010131	0.56	0.59	0.152
Rs7754840	0.33	0.32	0.559
Rs13266634	0.70	0.69	0.594
Rs564398	0.57	0.57	0.970
Rs10811661	0.83	0.82	0.473
Rs1111875	0.60	0.59	0.280
Rs7903146	0.27	0.27	0.816
Rs5219	0.35	0.37	0.409
Rs1495377	0.50	0.50	0.754
Rs8050136	0.38	0.37	0.771
Rs4430796	0.49	0.48	0.399
Rs757210	0.39	0.38	0.357
Mean number of risk alleles	14.5	14.5	0.977
(95% CI)	(14.4-14.6)	(14.3-14.7)	

Allele frequencies are reported for the T2D risk alleles. P-values report difference in genotype trend between offspring and partners and account for family relations among the offspring by using robust standard errors.

## DISCUSSION

The main findings of the present study are twofold. First, we were able to replicate the association of SNPs discovered by GWA's with T2D to associate with prevalence of diabetes and with glucose levels in the Leiden Longevity Study. Second, these polymorphisms did not differ in frequency or association with glucose levels between offspring of long-lived siblings and their partners.

Despite our relatively small cohort, we were able to confirm in our population that SNPs associated with T2D in GWA's also associate with prevalence of diabetes and levels of glucose in the Leiden Longevity Study. The effect sizes of associations with single SNPs identified by GWAs are generally low. Because our study population is relatively small to detect such small effect size we calculated the total number of risk alleles for each individual, to maximize power. This model assumes that there may be an additive effect of the SNPs. We then stratified the total study population in tertiles of the numbers of risk alleles. Our findings are in line with a recent publication [14], in which it was described that an increasing number of risk alleles was associated with an increased prevalence of diabetes.

We found no association of T2D risk alleles with familial longevity. Previously, in the same cohort we found the offspring to have better glucose tolerance than controls [5], which in clamp studies was concluded to result from differences in peripheral glucose disposure [15] In the present study we do replicate the association of the SNPs with glucose metabolism, yet there was no difference in allele frequency between offspring and controls. One possible explanation lies in the function of the selected SNPs. Ten of the fifteen selected SNPs are associated with alterations in beta cell response [16]. Although the main driver of T2D is peripheral insulin resistance, compromised beta cell function is believed to be an important (genetic) factor in the pathogenesis of T2D. The onset of type 2 diabetes occurs when beta-cell function cannot compensate for the high levels of insulin needed due to the peripheral insulin resistance. The fact that we find here that these SNPs do not account for the beneficial glucose handling in longevity is in line with our observation that beta cell function does not differ between partners and offspring in an oral glucose tolerance test [5]. The difference in glucose handling between offspring and controls might rather be determined by enhanced insulin sensitivity of the liver or peripheral tissues. Furthermore, in the same study population, we recently found no difference in disease risk allele frequencies between the long-lived parents of the offspring and younger controls [17]. This implicates that longevity that longevity is not compromised by risk alleles, but may rather be determined by protective alleles specific for longevity. Such mechanisms may for instance involve nutrient sensing pathways, such as mTOR, that affect insulin sensitivity [18]. Indeed, also in our study population, differential expression in mTOR signaling components was observed between offspring and controls in preliminary analyses [19]. More research is needed to elucidate the determinants of insulin sensitivity. Taken together these data suggest that SNPs that associate with T2D identified by GWA studies and that associate with beta cell function are not major determinants of the beneficial glucose tolerance that characterizes familial longevity.

## METHODS

### The Leiden Longevity Study

Nonagenarian sibling pairs were included when aged older than 89 years for men and 91 years for women and having at least one sister or a brother fulfilling these age criteria, who was also willing to participate. Because proper controls are lacking at very high ages, the offspring of the nonagenarian siblings were asked to be included in the study as well. The partners thereof were included in the study to serve as a control group, representing the general population at an age comparable to the offspring. The total study population, excluding the nonagenarian siblings, consisted of 2415 participants (1671 offspring; 744 partners). The Medical Ethical Committee of the Leiden University Medical Centre approved the study and informed consent was obtained from all participants.

### Phenotyping

Blood samples were taken at baseline for extraction of DNA and the determination of non-fasted serum parameters. Glucose and insulin were available for 2337 and 2287 participants respectively. We collected additional information and biomaterials from the generation of offspring and partners, including self-reported information on life style and bodily measures. Body height and weight were obtained from 1670 participants. Body mass index was calculated from these data. Information on medical history was requested from the participants' general practitioners. In a subgroup of 234 offspring and their partners we performed an oral glucose tolerance test. We calculated the area under the curve for each individual as a measure of glucose tolerance [5].

### Genotyping

*Selection of Polymorphisms.* We reviewed 266 GWAS that were published up to February 2009 (http://www.genome.gov/26525384), 10 of which reported on type-2 diabetes. These 10 GWAS reported 13 loci to be associated with type 2 diabetes (T2D) within at least two independent GWAS. To compile a set of disease risk alleles for each locus the most replicated SNP was selected and subsequently, in case of equal number of replications, the SNP with the lowest reported p-value. For two loci, one additional SNP was selected that was in low to moderate LD (r^2^ < 0.80) with the most replicated SNP. Thus 15 SNPs were selected for analysis, covering 13 loci (see Table 1).

*Genotyping.* These 15 SNPs were genotyped using Sequenom iPLEX. The average genotype call rate for these SNPs was 96.9% and the average concordance rate was 99.7% among 128 duplicated control samples. Complete genotyping of all 15 SNPs succeeded in 2090 participants (87%). Because in complex human traits, single mutations are not expected to have large effects and are therefore hard to identify [20], we calculated for each individual the total number of T2D risk alleles. Demographic and antropometric data, disease prevalence and levels of glucose and insulin were compared between these groups. Allele frequencies were comparable to those described in the literature and all genotype distributions were in Hardy-Weinberg equilibrium.

### Statistical Analysis

Continuous data were distributed normally except for levels of insulin, which was log transformed. Age, sex and disease prevalence frequencies are reported unadjusted. Means and 95% confidence intervals of antropometrics, glucose and insulin are reported adjusted for age and sex. Differences in disease prevalence between groups were assessed using logistic regression, adjusting for age and sex. Difference in antropometrics and levels of glucose and insulin were calculated using a linear regression model adjusting for age and sex. All p-values for differences between groups were adjusted for family relationships using robust standard errors, except for age and sex, which were calculated crude. Differences were considered significant when the p value was below 0.05. All statistical analyses were performed with STATA (version 10.0, USA) and SPSS (version 16.0, USA), and in all analyses we made use of robust standard errors to account for familial relationships among the offspring.
